# Global mapping of nonseismic sea level oscillations at tsunami timescales

**DOI:** 10.1038/srep40818

**Published:** 2017-01-18

**Authors:** Ivica Vilibić, Jadranka Šepić

**Affiliations:** 1 Institute of Oceanography and Fisheries, Šetalište I. Meštrovića 63, 21000 Split, Croatia

## Abstract

Present investigations of sea level extremes are based on hourly data measured at coastal tide gauges. The use of hourly data restricts existing global and regional analyses to periods larger than 2 h. However, a number of processes occur at minute timescales, of which the most ruinous are tsunamis. Meteotsunamis, hazardous nonseismic waves that occur at tsunami timescales over limited regions, may also locally dominate sea level extremes. Here, we show that nonseismic sea level oscillations at tsunami timescales (<2 h) may substantially contribute to global sea level extremes, up to 50% in low-tidal basins. The intensity of these oscillations is zonally correlated with mid-tropospheric winds at the 99% significance level, with the variance doubling from the tropics and subtropics to the mid-latitudes. Specific atmospheric patterns are found during strong events at selected locations in the World Ocean, indicating a globally predominant generation mechanism. Our analysis suggests that these oscillations should be considered in sea level hazard assessment studies. Establishing a strong correlation between nonseismic sea level oscillations at tsunami timescales and atmospheric synoptic patterns would allow for forecasting of nonseismic sea level oscillations for operational use, as well as hindcasting and projection of their effects under past, present and future climates.

Knowledge of sea level extremes in changing climates is of the utmost importance, as sea level extremes inevitably impact densely populated coastlines^1^. A broad range of processes spanning a wide range of spatial and temporal scales contribute to these extremes^3^,^4^,^5^, from long-term trends and variability, seasonal changes, planetary and synoptic sea level oscillations, up to the mesoscale. Processes operating at most of these scales have been fairly well documented through global and regional assessment of long-term sea level and remote sensing records^6^,^7^,^8^, as standard tide gauge outputs are available at hourly resolution in global datasets such as the one managed by the University of Hawaii (http://uhslc.soest.hawaii.edu), and remote sensing data have been available since 1993 (http://www.aviso.altimetry.fr). Still, a number of high-frequency processes that substantially contribute to sea level extremes (such as tsunamis, meteotsunamis, and infragravity waves) cannot be properly assessed using hourly data. The science of tsunamis, due to their global importance and impact, has constantly been at the forefront of sea level research^9^, while the remainder of the high-frequency sea level signal, which can be defined as consisting of nonseismic sea level oscillations at tsunami timescales (NSLOTT), has been researched only locally^10^,^11^,^12^,^13^. The concept of NSLOTT events refers to all sea level oscillations except tsunamis that appear at periods between a few minutes to a few hours. Extreme realizations of NSLOTT, which are capable of producing damage or impacts in a region, are referred to as meteorological tsunamis or meteotsunamis^10^. No global study assessing the importance of NSLOTT events has been conducted so far, while there are several review papers documenting the world distribution of known meteotsunamis^10^,^14^.

Recently, after the 2004 Sumatra-Andaman tsunami^15^, the Intergovernmental Oceanographic Commission (IOC) coordinated efforts to upgrade sea level observations to minute timescales and to create a global operational database called the Sea Level Station Monitoring Facility (http://www.ioc-sealevelmonitoring.org). This database contains raw, not-quality checked data with a temporal resolution of 1 min, and it is the only global database containing multi-year global sea level observations at tsunami timescales. An initial regional (Mediterranean) study using these data showed that NSLOTT events may be dominant over tides during extreme sea level episodes and should thus be included in any sea level assessment^13^.

The global distributions of NSLOTT variance and maximum range, in terms of their absolute values and relative to total sea level variance and range, lead to similar conclusions (Fig. 1). Variance is generally not high, up to 2 cm^2^, being highest in shelf areas such as the North Sea. However, the tides are also large in the North Sea^16^; therefore, NSLOTT contribution to overall sea level variance is less than 0.25% there. Moreover, NSLOTT contribution to overall sea level variance is not high globally; this contribution is up to 1.25% in low-tidal basins such as the Mediterranean and is even lower throughout rest of the World Ocean.

NSLOTT events are, however, highly changeable in time. During the most extreme episodes, they may range up to a metre in some basins, such as the North Sea and the South Atlantic, and even more at specific stations. The maximum station-averaged range of NSLOTT is greater than 35 cm in all coherent regions of the World Ocean, reaching average values up to 85 cm in some basins. The contributions of NSLOTT events to the overall maximum sea level range may be as high as 50% in low-tidal areas (such as the Mediterranean and the Caribbean), surpassing the tidal range. These oscillations thus have the potential to strongly affect coastlines, especially when they are combined with strong currents, which can be an order of magnitude stronger at tsunami than at tidal frequencies^17^.

Interestingly, the absolute values of NSLOTT variance and range (right-hand panels in Fig. 1) show a zonal pattern that shows generally lower values in the tropical and subtropical regions and increases towards the poles. Averaging over 10° zonal belts confirms this (Fig. 2); the average NSLOTT variance and range are two to three times lower between −20°S and 20°N than in the mid-latitudes. Zonal distributions of variance and range are correlated at the 90 and 99% levels, respectively, with zonal average wind speed at the 500-hPa isobar (which is located in the middle troposphere at a height of approximately 5.5 km). The former correlation significance increases to the 99% level when only data from 50°S-50°N are analysed. This indicates a lower resemblance between NSLOTT and zonal mid-tropospheric winds at the higher mid-latitudes (Fig. 2), where a decrease in mid-tropospheric winds is not followed by a decrease in the NSLOTT variance. The importance of mid-tropospheric winds in generating NSLOTT events has been recognized in a number of studies^13^,^18^,^19^, which show that 500-hPa winds (at the level of the mid-tropospheric jet) are exceptionally strong during NSLOTT events.

The anomalously strong 500-hPa winds during NSLOTT events are known to co-occur with other patterns, including (i) instability of the mid-troposphere jet, (ii) winds that quasi-linearly decrease towards the surface, (iii) a poleward flow of warm and dry air in the lower troposphere, and (iv) a weak surface cyclone barely detectable in surface air pressure charts. These patterns have so far been recognized in a few regional studies^18^,^19^,^20^,^21^. To check for the existence of globally dominant NSLOTT synoptic patterns, we extracted the most extreme NSLOTT episodes (in terms of their maximum range) from a number of representative stations with multi-annual high-quality sea level series and related them to the ECMWF (European Centre for Middle-range Weather Forecasts) ERA-Interim reanalysis fields. Figure 3 displays the atmospheric fields that are found to be characteristic for NSLOTT events and averaged over the 15 strongest NSLOTT episodes in four selected areas. The maximum/median wave heights measured during these events at Esperance (Australia), Nagasaki (Japan), Clearwater Beach (the Gulf of Mexico), and Bahia Mansa (Chile) stations were equivalent to 101/70, 107/74, 93/40 and 152/99 cm. These events did not cause any reported damage, even though they constituted at most 50, 34, 40 and 42% of the entire sea level range measured at these stations, respectively. However, because they were not superimposed on extreme surges, they did not flood the coastal area and infrastructure adapted to such sea level changes. However, currents may be quite strong during meteotsunami events^10^,^18^ and normally affect the safety of navigation and harbour infrastructure^14^,^22^.

The synoptic patterns over three of the chosen areas are similar to conditions during previously described NSLOTT events, while the synoptic patterns over Chile stand out as somewhat different. During most of the NSLOTT episodes above southeastern Australia, Japan and the Gulf of Mexico, (i) weak to moderate pressure minima are located above the stations affected. These minima are accompanied by (ii) poleward advection of warm air in the lower troposphere (at a pressure level of ~850 hPa) and (iii) strong mid-tropospheric jets (speeds of 26–32 m/s at pressure levels of ~500 hPa) embedded in (iv) unstable atmospheric layers (Ri < 0.25 at pressure levels of ~400–700 hPa).

A common source mechanism associated with such synoptic patterns is known to be responsible for NSLOTT and meteotsunami events in the Mediterranean^15^,^20^. This mechanism is called *wave ducting*^23^,^24^ and is associated with the generation and horizontal propagation of atmospheric gravity waves in the lower troposphere. These atmospheric waves are often generated by mid-tropospheric shear^25^ and can be quite strong below the unstable mid-tropospheric jet. They are further capped in the stable lower troposphere by the unstable jet and are detectable at the ocean surface as pronounced air pressure disturbances. The propagation velocity of these pressure disturbances commonly matches the jet velocity^23^,^24^.

When the speed of atmospheric disturbances matches the speed of long ocean waves, the resonant transfer of energy from the atmosphere to the ocean occurs through a phenomenon called the *Proudman resonance*^26^. The speed of long ocean waves equals 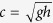, where *g* is gravity acceleration and *h* is water depth. We hypothesize that Proudman resonance occurs during most of the NSLOTT events analysed in this paper. For example, the mid-tropospheric jet speed of 28–32 m/s found during the Esperance and Nagasaki NSLOTT events (Fig. 3) is equivalent to the speed necessary for resonant generation of ocean waves at depths between 80 and 100 m. These depths encompass large portions of the 100-km wide shelf to the southwest of the Esperance station, as well as the 700-km wide shelf to the southeast of Nagasaki station stretching into the East China Sea. It should be noted that the average speed of the mid-tropospheric jet over Japan matches the speed of the atmospheric disturbance (~ 31 m/s) that caused the destructive 1979 ‘abiki’ event (the local Japanese name for meteotsunamis)^10^,^27^. The slightly lower jet speed (26–28 m/s) documented at the Clearwater Beach station (Fig. 3c) also matches the depth distribution (below 100 m) along the 200-km wide western Florida shelf.

However, the atmospheric patterns related to strong NSLOTT events differ in some world regions, like the Chilean coast (Fig. 3d), where these events are associated with strong meridional gradients in air pressure. Indeed, intense high-frequency air pressure oscillations may be generated also by the wave-CISK (Convective Instability of the Second Kind) mechanism, squall lines, frontal passages or other phenomena^28^,^29^. Some of these processes are presumably more relevant at the higher mid-latitudes where deep and energetic extratropical cyclones that stretch over the whole troposphere normally form^30^.

This analysis indicates a relationship between a certain atmospheric setup and NSLOTT events in an area. However, quantification of this relationship is a challenge, requiring synthesis of the atmospheric setup into a simple index or indices, which can then be correlated to the NSLOTT intensity. Such an analysis will allow assessment of whether NSLOTT events commonly result from a specific atmospheric setup or not. A template for this analysis might be the recently introduced meteotsunami index for the area surrounding the Balearic Islands^31^. This index is a linear combination of a number of atmospheric variables that are highly correlated with high-frequency sea level oscillations measured at a single station.

To conclude, our analysis shows that (i) NSLOTT events constitute an important part of the sea level budget in the World Ocean and are especially important during extreme episodes, and (ii) NSLOTT events are strongly associated with specific atmospheric patterns, suggesting that the wave-ducting mechanism is responsible for generation of tsunamigenic atmospheric disturbances within selected areas of the World Ocean. Several important implications emerge from this study. First, NSLOTT events should be taken into account in any sea level assessment study, particularly when assessing sea level extremes. These studies also need to include an assessment of other phenomena that contribute to extreme high-frequency sea level oscillations, such as (i) tsunamis, whose relevance varies strongly between different oceans, areas and basins^32^, and (ii) tropical cyclones, which may cause extraordinary high-frequency sea level oscillations and primarily affect low-lying areas within the tropics^33^, where NSLOTT oscillations are at a minimum. Second, the global tide gauge network should be standardized to sample at the minute resolution, with quality check procedures implemented before releasing data to the public, as a large number of stations provide problematic data that are corrupted by spikes, offsets, drifts in time and space, scaling changes, etc. Finally, the demonstrated connection between NSLOTT events and atmospheric patterns may be used for NSLOTT forecasting and reconstruction under past, present and future climates and for identifying site-specific correlations between NSLOTT and atmospheric reanalysis patterns, which can later be used to forecast NSLOTT events a week in advance using operational forecasts (like those of the ECMWF) or to reconstruct them using climate model output.

## Methods

We downloaded high-frequency sea level data from the Intergovernmental Oceanographic Commission (IOC) Sea Level Station Monitoring Facility website at http://www.ioc-sealevelmonitoring.org. Stations having at least a 12-month long time series and containing less than 10% data gaps were used in our analyses. Time series with predominantly bad records, as determined using visual inspection, are not considered. The dataset passed into the analyses included 336 sea level time series sampled with 1-min resolution. Selected time series passed a rigorous quality control, which included automatic despiking procedures and manual control for spikes, shifts, drifts, changes in tidal range and other changes. Data related to tsunami events listed in tsunami catalogues (NGDC/WDS Global Historical Tsunami Database) were omitted from the analyses. The series were then filtered with a 2-h Kaiser-Bessel high-pass filter and grouped into coherent regions.

Fifteen NSLOTT events with the largest wave heights were extracted from selected stations and associated with atmospheric patterns taken from ERA-Interim reanalysis data products at the nearest times. ERA-Interim reanalysis data products are available from the ECMWF (European Centre for Middle-range Weather Forecast) website at www.ecmwf.int and have a temporal resolution of 6 h. The mean sea level pressure, the temperature at the 850-hPa level, the wind at the 500-hPa level and the Richardson number Ri were used in the analysis, as these variables have been found important in meteotsunami studies^18^,^19^,^20^,^21^,^24^. The Richardson number is a measure of the stability of the atmospheric layer and is computed from the Brunt–Väisälä frequency N (s^−2^), the wind speed u (m/s) and the height z (m) as Ri = N^2^/(du/dz)^2^. The mid-troposphere layer (400–700 hPa) at a particular ERA-Interim grid point was considered unstable if Ri < 0.25.

Finally, zonally averaged global winds at the 500-hPa level were computed from the whole ERA-Interim dataset (1979–2015).

## Additional Information

**How to cite this article**: Vilibić, I. and Šepić, J. Global mapping of nonseismic sea level oscillations at tsunami timescales. *Sci. Rep.* **7**, 40818; doi: 10.1038/srep40818 (2017).

**Publisher's note:** Springer Nature remains neutral with regard to jurisdictional claims in published maps and institutional affiliations.

## Figures and Tables

**Figure 1:**
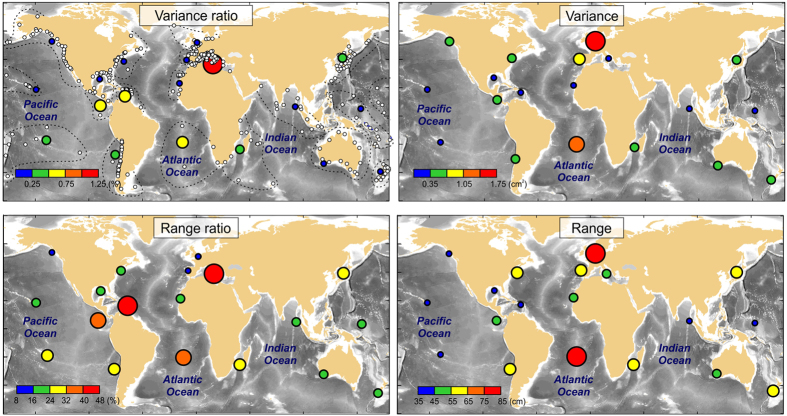
Ratio between NSLOTT (sea level oscillations at periods lower than 2 h) and total sea level variances (*upper left*) and their maximum ranges (*lower left*), as well as NSLOTT absolute variance (*upper right*) and maximum ranges (*lower right*), all averaged over coherent areas. Tide gauge stations used in the analyses are marked by white circles in the upper left panel, which also shows borderlines separating different areas. Figure was created using MATLAB R2014a software (http://www.mathworks.com).

**Figure 2:**
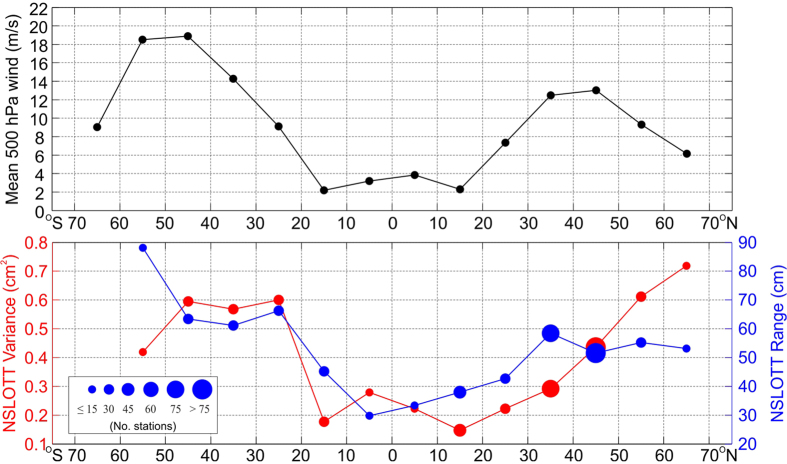
*Upper*: mean wind speeds at 500-hPa level obtained from the ECMWF ERA-Interim reanalysis (1979–2016) and averaged over 10° zonal belts; *lower*: NSLOTT variances and maximum ranges averaged over 10° zonal belts. The size of each circle is proportional to the number of tide gauge stations in the respective zonal belt. Figure was created using MATLAB R2014a software (http://www.mathworks.com).

**Figure 3:**
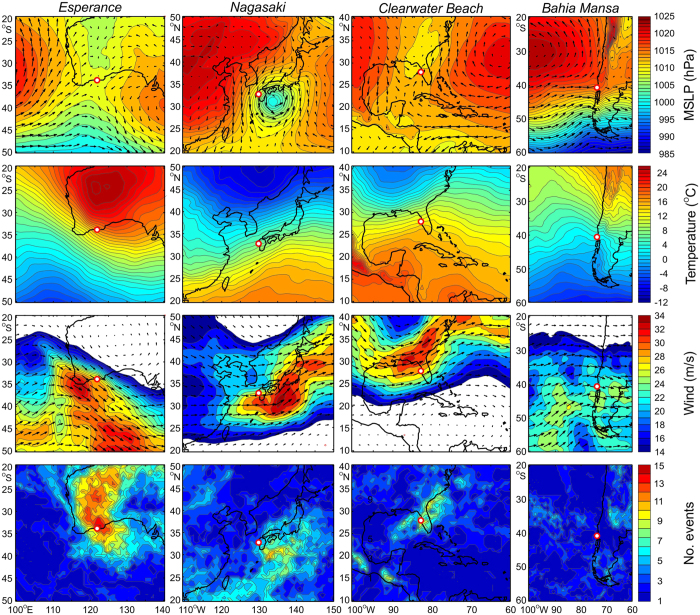
*From top to bottom*: mean sea level pressure (MSLP) and surface winds; temperature at 850-hPa level; winds at 500-hPa level; number of times during which the minimum Richardson number between 400 and 700 hPa was lower than 0.25 at each grid point; all averaged (counted) over the 15 strongest NSLOTT events at (*from left to right*) Esperance (Australia), Nagasaki (Japan), Clearwater Beach (the Gulf of Mexico), and Bahia Mansa (Chile) stations. Figure was created using MATLAB R2014a software (http://www.mathworks.com).
